# Steroid profile in patients with breast cancer and in mice treated with mifepristone

**DOI:** 10.1530/ERC-23-0238

**Published:** 2023-12-13

**Authors:** Andres Elía, Leo Saldain, Silvia Lovisi, Paula Martínez Vazquez, Javier Burruchaga, Caroline A Lamb, Isabel Alicia Lüthy, Federico Diez, Natalie Z M Homer, Ruth Andrew, Paola Rojas, Claudia Lanari

**Affiliations:** 1 Instituto de Biología y Medicina Experimental (IBYME), CONICET, Argentina; 2 Hospital de Agudos “Magdalena V de Martínez”, General Pacheco, Argentina; 3 University/BHF Centre for Cardiovascular Science, Queen's Medical Research Institute, University of Edinburgh, UK

**Keywords:** mifepristone, progesterone receptor isoforms, breast cancer treatment, window of opportunity trial, steroid hormone levels, plasma, mice, antiprogestins

## Abstract

Progesterone receptors (PRs) are biomarkers used as prognostic and predictive factors in breast cancer, but they are still not used as therapeutic targets. We have proposed that the ratio between PR isoforms A and B (PRA and PRB) predicts antiprogestin responsiveness. The MIPRA trial confirmed the benefit of 200 mg mifepristone, administered to patients with tumors with a high PRA/PRB ratio, but dose-ranging has not been conducted. The aim of this study was to establish the plasma mifepristone levels of patients from the MIPRA trial, along with the resultant steroid profiles, and compare these with those observed in mifepristone-treated mice using therapeutic schemes able to induce the regression of experimental mammary carcinomas with high PRA/PRB ratios: 6 mg pellets implanted subcutaneously, or daily doses of 12 mg/kg body weight. The plasma levels of mifepristone and other 19 plasma steroids were measured by liquid chromatography–tandem mass spectometry. In mifepristone-treated mice, plasma levels were lower than those registered in mifepristone-treated patients (i.e. day 7 after treatment initiation, pellet-treated mice: 8.4 ± 3.9 ng/mL; mifepristone-treated patients: 300.3 ± 31.7 ng/mL (mean ± s.d.; *P* < 0.001)). The increase in corticoid related steroids observed in patients was not observed in mifepristone-treated mice. The increase in progesterone levels was the most significant side effect detected in mifepristone-treated mice after 14 or 21 days of treatment, probably due to an ovarian compensatory effect not observed in postmenopausal patients. We conclude that in future clinical trials using mifepristone, the possibility of lowering the standard daily dose of 200 mg should be considered.

## Introduction

The idea that targeting the progesterone receptor (PR) in breast cancer could be a therapeutic approach has been considered for decades (reviewed in Abulafia *et al.* (1999), Lanari *et al.* (2012), Giulianelli *et al.* (2021)). Moreover, attempts had been performed using progestins such as medroxyprogesterone acetate (MPA) which was given in doses of up to 1 g daily (Pannuti *et al.* 1988) or antiprogestins such as mifepristone (Perrault *et al.* 1996), onapristone (Robertson *et al.* 1999, Cottu *et al.* 2018), and, more recently, telapristone acetate (Lee *et al.* 2019). Although partial responses were observed in several patients, the results obtained were less encouraging than originally expected (Lanari *et al.* 2012).

The MIPRA trial was launched following the hypothesis that the PR isoform ratio is crucial to predict the antiprogestin responsiveness of luminal breast carcinomas. This concept was based on previous basic research, mainly reported by the Edwards (Beck *et al.* 1993) and Horwitz (Tung *et al.* 1993) laboratories, who demonstrated that mifepristone acted as a progesterone agonist regulating gene expression in cells expressing PR isoform B (PRB). Along the same line, we have shown that antiprogestin-responsive tumors from the murine MPA-induced breast cancer model had higher levels of PR isoform A (PRA) than PRB (Helguero *et al.* 2003), and when they acquired antiprogestin resistance there was a change in the PR isoform ratio toward B and thus a potential switch from mifepristone acting as an antagonist to an agonist (Helguero *et al.* 2003, Wargon *et al.* 2009, 2011, 2015). In constitutive resistant tumors this change in ratio was due to selective PRA promoter methylation (Wargon *et al.* 2011), and in acquired resistant tumors the mechanism is still under investigation. Preclinical studies using *ex vivo* human breast tissue cultures and cell derived xenograft studies confirmed the murine data (Rojas *et al.* 2017). Encouragingly, results of the MIPRA window of opportunity study revealed a reduction of the proliferation index in 70% of tumors in patients with higher levels of PRA than PRB receiving 200 mg of mifepristone tablets *per* *os* for 14 days (Elia *et al.* 2022). This dose was selected since it was already proven to be effective and safe for the treatment of meningiomas over long periods of time (Grunberg *et al.* 2006, Ji *et al.* 2015, Karena *et al.* 2022).

Many questions arose after analyzing the MIPRA data, one being whether the dose selected was the most appropriate to treat breast cancer patients. Although mild side effects were observed in treated patients (Elia *et al.* 2022), this agent would be potentially administered together with antiestrogenic compounds, and lower doses may be safer for the patients in the long term, if still efficacious. To address this issue, we decided to compare the mifepristone plasma concentrations of mice receiving mifepristone schedules able to induce complete mammary tumor regression with those detected in mifepristone-treated patients, and in addition, to compare the pharmacodynamic changes in the concentration of the steroids related to the biosynthesis of corticoid/sex steroids. These data might then inform researchers whether lower doses could be assessed in the clinical setting.

## Materials and methods

### Study design

The MIPRA study was an open-label, interventional, prospective, single-arm clinical trial (ClinicalTrials.gov identifier: NCT02651844). The main goal was to evaluate the effect of mifepristone in postmenopausal breast cancer patients with higher levels of PRA than PRB, naïve from any other treatment. The primary outcome was to compare the proliferation index pre-treatment and post treatment. The trial included patients who spontaneously attended the Magdalena V. de Martinez Hospital at General Pacheco, Buenos Aires. Eligibility criteria included (i) postmenopausal status more than 1 year after the last menses, (ii) tumors larger than 15 mm, (iii) PRA/PRB ratios higher than 1.5 determined by Western blot, (iv) PR total levels ≥50% evaluated by immunohistochemistry, (v) World Health Organization condition of 1 with the adequate function of organs and systems: absolute neutrophil count 1500/mL; platelets ≥100,000/mL; hemoglobin ≥10 g/dL; CD4 count ≥400; creatinine <1.5 mg/dL; total bilirubin, aspartate aminotransferase, and alanine aminotransferase <1.5 U/L × upper limit of institutional normal. Patients were excluded if they (i) received any other treatment for cancer, (ii) had hepatitis or human immunodeficiency virus infection, (iii) had cognitive alterations that limited their understanding of the protocol, (iv) experienced a prolonged QT/QTc basal interval, or (v) had asthma or other autoimmune diseases. Twenty patients that met these criteria were treated with daily doses of 200 mg mifepristone administered as tablets (Abortab, Pharma-web; Canada) per os during the morning for 14 days.

### Studies with patients’ plasma

**Figure 1:**
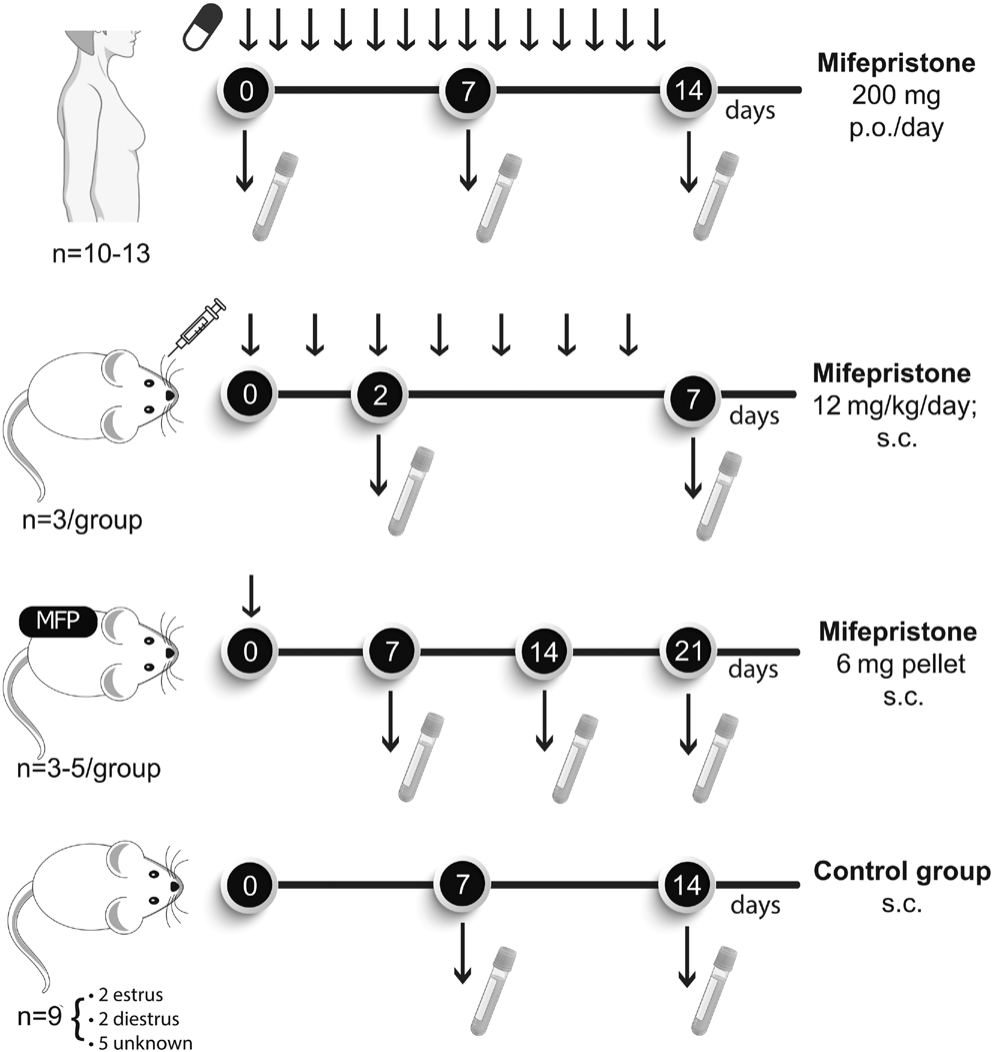
Scheme illustrating mifepristone administration and plasma collection in patients from the MIPRA trial and in mifepristone-treated mice.

Plasma samples were obtained before (*n* = 14) or after 7 (*n* = 16) or 14 (*n* = 13) days of mifepristone treatment (Fig. 1, top). In all cases, the blood was obtained prior to the mifepristone tablet administration. Nine of these patients completed the entire schedule, whereas in others, one of the plasma samples was missing. Plasma samples were collected in Vacutainer tubes and kept at −20 or −80°C until the study was ended. Aliquots of 200 μL were sent to the Mass spectrometry core of the Edinburgh Clinical Research Facility, University of Edinburgh. The LC system was a Water I Class, mobile phase A of 50 mM ammonium fluoride and mobile phase B of 50 mM ammonium fluoride in methanol: Column Kinetex C18 (150 × 2.1 mm; 2.6 μm): Mass Spect: Qtrap 6500+. Mifepristone together with other 19 steroids was measured using a supported liquid extraction method and LC-MS/MS (Denham 2020). The lowest and upper levels of detection, as well as the normal plasma concentration of each steroid, are shown in Supplementary Table 1 (see section on supplementary materials given at the end of this article).

## Mouse studies

### Animals

Two-month-old virgin female BALB/c mice (IBYME Animal Facility) were used. All animals were fed *ad libitum* and kept in air-conditioned room at 20 ± 2°C with a 12-h light–12-h darkness cycle period.

### Murine mammary carcinomas

Murine mammary carcinomas from the MPA-induced breast cancer model were used (Lanari *et al.* 2009). The murine mammary carcinoma 59-2-HI, which expresses high estrogen receptor alpha (ER) and PR levels, regresses completely when a 6 mg mifepristone pellet is implanted subcutaneously (Vanzulli *et al.* 2002). Tumors were transplanted into BALB/c mice by trocar and, when they reached a size of 5 mm in the long axis, an MFP pellet (6 mg) was implanted subcutaneously as described (Sahores *et al.* 2013). Tumors were measured twice a week with a Vernier caliper to assess the functionality of pellets.

### Mouse plasma

Adult female mice were implanted with mifepristone (6 mg) pellets, or were sham-treated with Silastic pellets without hormones. Mice were bled by cardiac puncture 1 (*n* = 5), 2 (*n* = 3), or 3 (*n* = 3) weeks after pellet implantation, and plasma samples prepared and stored at −80°C. Control mice were bled 1 (*n* = 2) or 2 (*n* = 3) weeks after empty pellet implantation (Fig. 1, bottom). In addition, further mice were treated with daily injections of mifepristone (12 mg/kg body weight) subcutaneously, a treatment protocol that also induces the regression of this tumor variant (Wargon *et al.* 2009). Mice were bled 48 h or 7 days after treatment initiation (*n* = 3/group). In both dosing scenarios, mice were bled 24 h after drug inoculation. Plasma from three untreated mice were used as controls. From the nine control mice, two were in estrus and two in diestrus. In the other 5 control mice, the estrous cycle was not determined. Samples were sent to the Mass spectrometry Core of the Edinburgh Clinical Research Facility, as explained earlier, to measure the levels of mifepristone and other plasma steroids.

### Statistical analysis

Nonmatched plasma levels were compared using nonparametric Kruskal–Wallis tests and Dunn’s multicomparison *post* *hoc* tests. For comparison of plasma levels from the same individual, data were analyzed with nonparametric test for matched data (Friedman test). Tumor growth curves were compared using two-way ANOVA and Tukey post *t*-tests.

### Ethics statement

A written informed consent was obtained from all patients. This MIPRA trial was conducted in accordance with the Declaration of Helsinki and approved by the Institutional Review Boards of the hospital and IBYME (2012-026). Mouse experiments were approved by the IBYME-CONICET Institutional Animal Care and Use Committee authorities (032-2018) and complied with standards of animal ethics. The approval included the experiments reported herein and others that were not included in this article.

## Results

### Mifepristone levels in plasma from mifepristone-treated mice as compared with those of mifepristone-treated patients

**Figure 2:**
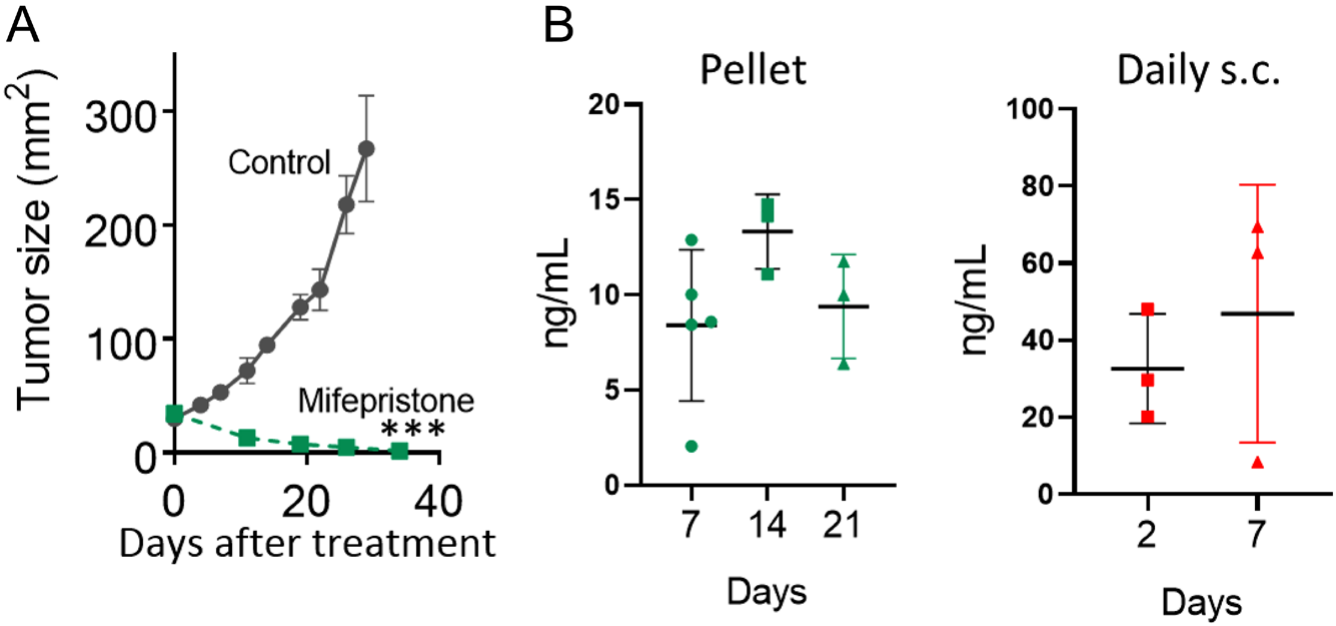
Mifepristone plasma levels capable of inducing complete regression of a mouse mammary carcinoma with PRA levels higher than PRB. (A) Tumor growth curves illustrating 59-2-HI tumor regression after administering a 6 mg mifepristone pellet subcutaneously at day 0; (*n* = 5); (B) Mifepristone plasma levels (mean ± s.d.) after 7, 14, or 21 days after pellet implantation (left) or after 48 h or 7 days of daily doses of 12 mg/kg body weight (right). A full color version of this figure is available at https://doi.org/10.1530/ERC-23-0238.

Mifepristone administered as pellets (6 mg; Fig. 2A) or as subcutaneous daily doses of 12 mg/kg body weight (Wargon *et al.* 2009) induced complete regression of ER+ PR+ mammary carcinomas with higher levels of PRA than PRB (Lanari *et al.* 2009). Thus, our first goal was to compare the mifepristone plasma levels in these tumor models in which we already know that PR are involved in their growth (Lamb *et al.* 1999, 2005) with those observed in mifepristone-treated patients.

As observed in Fig. 2B left, plasma mifepristone levels remained almost constant during 21 days after pellet implantation. Plasma levels were more heterogeneous following daily injections (Fig. 2B right; i.e. 7 days pellet: (mean ± s.d.) 8.4 ± 3.9 ng/mL, 21.5 ± 9.1 nmol/L (*n* = 5); 7 days daily doses: (mean ± s.d.) 46.9 ± 33.4 ng/mL, 109 ± 77 nmol/L (*n* = 3)). Despite these differences, both doses induced complete tumor regression. In patients, significant differences were not observed between mifepristone levels after 1 or 2 weeks of treatment (day 7 (mean ± s.d.): 300.3 ± 31.7 ng/mL (690 ± 72.9 nmol/L); day 14: 320 ± 54.3 ng/mL (745 ± 126.4 nmol/L; *n* = 11); (Elia *et al.* 2022)), and they were more than twenty times higher than those observed in the animal model using mifepristone pellets (*P* < 0.001)**,**suggesting that the effect of lower mifepristone doses in breast cancer patients with higher levels of PRA than PRB should be tested.

### Pharmacodynamic responses: steroid plasma levels in breast cancer patients or mice treated with mifepristone

Since mifepristone at high concentrations exerts antiglucocorticoid (Gaillard *et al.* 1984) and antiandrogenic (Song *et al.* 2004) effects, we decided to compare the plasma steroid profile of mifepristone-treated breast cancer patients with that of mifepristone-treated mice.

**Figure 3:**
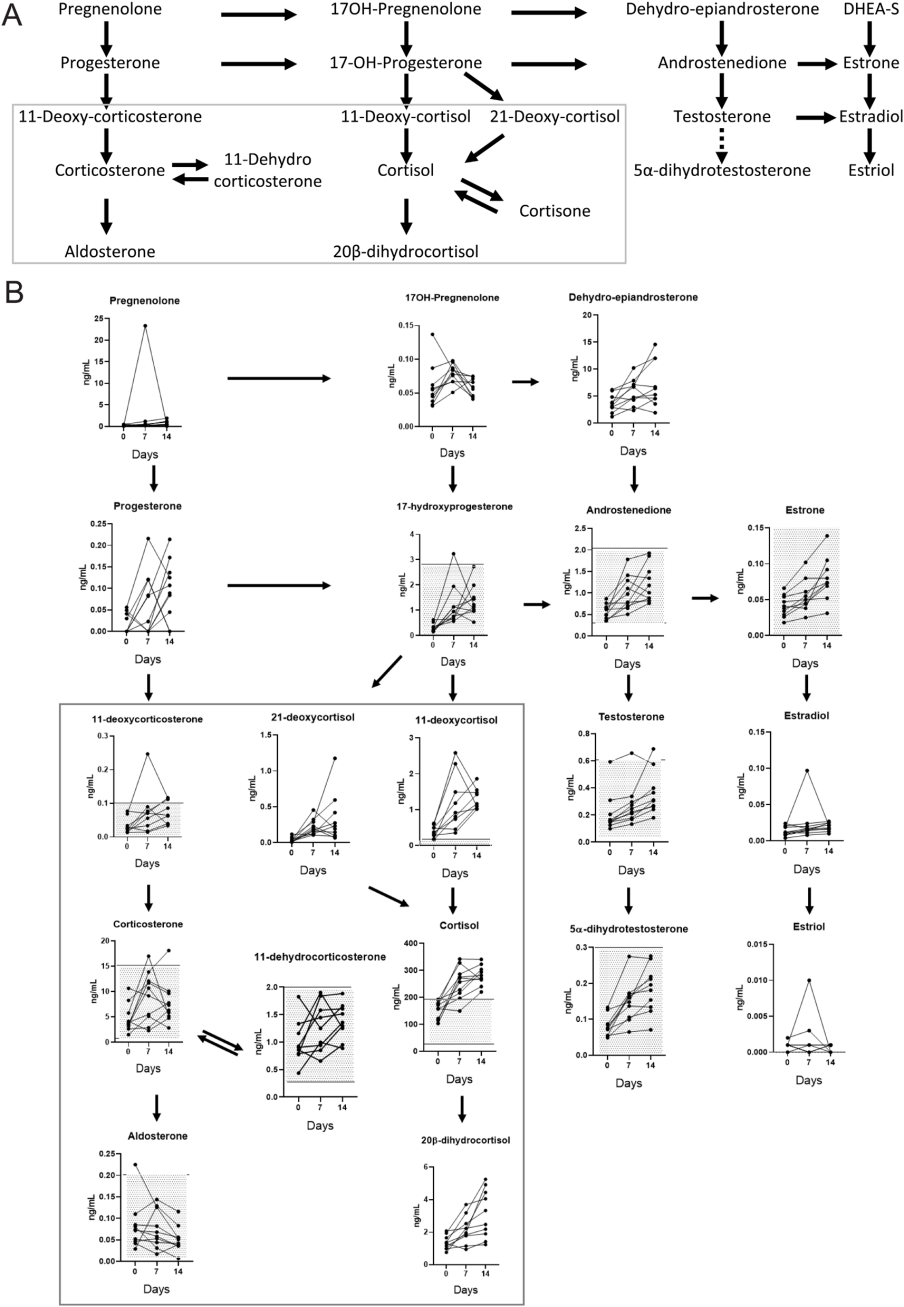
Changes in steroid hormone levels in postmenopausal breast cancer patients after mifepristone treatment. (A) Human steroidogenesis pathway. (B) Profile of 19 steroid hormones evaluated in plasma by liquid extraction and LC-MS/MS before and after 7 or 14 days of mifepristone treatment. Values that fall in dotted areas are within normal levels detected in pre- or postmenopausal women. In all cases there was a significant change after treatment. Data were analyzed using a nonparametric analysis for matched samples (Friedman test). In most cases, except for cortisol, the changes were within physiological levels. The data of nine patients are shown. The graphs are arranged according to the pathways shown in A.

The plasma levels of steroids related to the corticoid, androgenic, and estrogen biosynthesis registered in patients before, and after 7 or 14 days of treatment are shown in Table 1. Significant variations were observed in 17 out of 19 steroids tested; however, most of them remained within physiological levels detected in pre- or postmenopausal women (Supplementary Table 1), except for cortisol, which surpassed the normal levels reaching a mean value of 350 ng/mL at day 14 after treatment, and testosterone, in which 2 values surpassed the upper limit. Changes were not observed in aldosterone and progesterone levels, and those in estrogenic compounds were small, although significant. The individual shifts of each of the 9 patients in which the three measurements were performed is shown in Fig. 3.

**Table 1:** Steroid hormones in postmenopausal breast cancer patients untreated or treated with mifepristone for 7 or 14 days.

Steroid	Pre-treatment (nmol/L) *n* = 14		Mifepristone, day 7 (nmol/L) *n* = 16		Mifepristone, day 14 (nmol/L) *n* = 13		*P*^a^
	Median (range)	*X̅* ± S.D.	Median (range)	*X̅* ± S.D.	Median (range)	*X̅* ± S.D.	
Pregnenolone	0.62 (0.01–1.5)	0.7 ± 0.5	0.86 (0.09–73.9)	5.7 ± 18.2	1.6 (0.23–6)	2.2 ± 1.8	*P* < 0.05
11-dehydrocorticosterone	2.4 (1.2–5.3)	2.6 ± 1	3.7 (1.9–5.5)	3.7 ± 1.1	3.9 (2.3–5.5)	3.8 ± 0.9	*P* < 0.01
11-deoxycorticosterone	0.08 (0.03–0.2)	0.03 ± 0.06	0.2 (0.04–0.8)	0.2 ± 0.2	0.2 (0.07–0.4)	0.2 ± 0.1	*P* < 0.01
11-deoxycortisol	1.1 (0.5–1.8)	1.2 ± 0.5	3.2 (1–9)	4.0 ± 2.5	3.2 (1.5–5.8)	3.5 ± 1.2	*P* < 0.001
17-hydroxyprogesterone	1.01 (0.5–37.5)	3.6 ± 9.7	3.2 (1.3–54.6)	7.3 ± 13	3.7 (1.6–9)	4.3 ± 2.2	*P* < 0.001
20beta-dihydrocortisol	3 (2–5.7)	3.4 ± 1.1	5.2 (2.2–10.3)	5.8 ± 2.6	6.8 (3.4–24.5)	9.1 ± 6.1	*P* < 0.001
21-deoxycortisol	0.08 (0.02–10.4)	0.8 ± 2.7	0.6 (0.1–8.6)	1.2 ± 2	0.6 (0.19–4.7)	1.2 ± 1.4	*P* < 0.001
Aldosterone	0.19 (0.02–0.5)	0.19 ± 0.1	0.2 (0.05–0.4)	0.2 ± 0.1	0.1 (0.02–0.3)	0.1 ± 0.09	ns
Androstenedione	2.2 (1.2–5.3)	2.2 ± 1.1	3.6 (1.5–10.6)	4.5 ± 2.7	4.5 (2.6–6.7)	4.1 ± 1.4	*P* < 0.001
Corticosterone	9.8 (4.3–30.8)	11.9 ± 7.4	27.1 (6.6–49.2)	25.7 ± 14.1	21.2 (8.2–52.4)	21.7 ± 11.2	*P* < 0.01
Cortisol	447 (268–530)	425 ± 89	730 (413–1014)	712 ± 199	747 (493–1215)	780 ± 176	*P* < 0.001
Cortisone	65 (47–96)	66 ± 13	74 (51–130)	76 ± 17	80 (70–82)	82 ± 10	*P* < 0.01
Dehydroepiandrosterone	10.6 (4–21)	12 ± 5	19 (8–35)	20 ± 8	22 (7–51)	24 ± 13	*P* < 0.01
Dihydrotestosterone	0.26 (0.16–0.46)	0.3 ± 0.09	0.5 (0.22–0.95)	0.5 ± 0.2	0.62 (0.2–1)	0.6 ± 0.2	*P* < 0.001
Estradiol	0.05 (0.01–0.09)	0.05 ± 0.02	0.06 (0.03–0.4)	0.08 ± 0.07	0.08 (0.03–0.1)	0.07 ± 0.02	*P* < 0.05
Estrone	0.14 (0.06–0.24)	0.15 ± 0.05	0.2 (0.09–0.5)	0.2 ± 0.1	0.28 (0.1–0.51)	0.28 ± 0.1	*P* < 0.01
Progesterone	0.13 (0.09–1.3)	0.3 ± 0.4	0.3 (0.07–2.1)	0.6 ± 0.6	0.4 (0.1–1)	0.5 ± 0.3	ns
Testosterone	0.56 (0.34–2)	0.7 ± 0.4	1 (0.5–2.8)	1.2 ± 0.7	1 (0.6–2.4)	1.2 ± 0.5	*P* < 0.01

^a^Kruskal–Wallis test.

**Figure 4:**
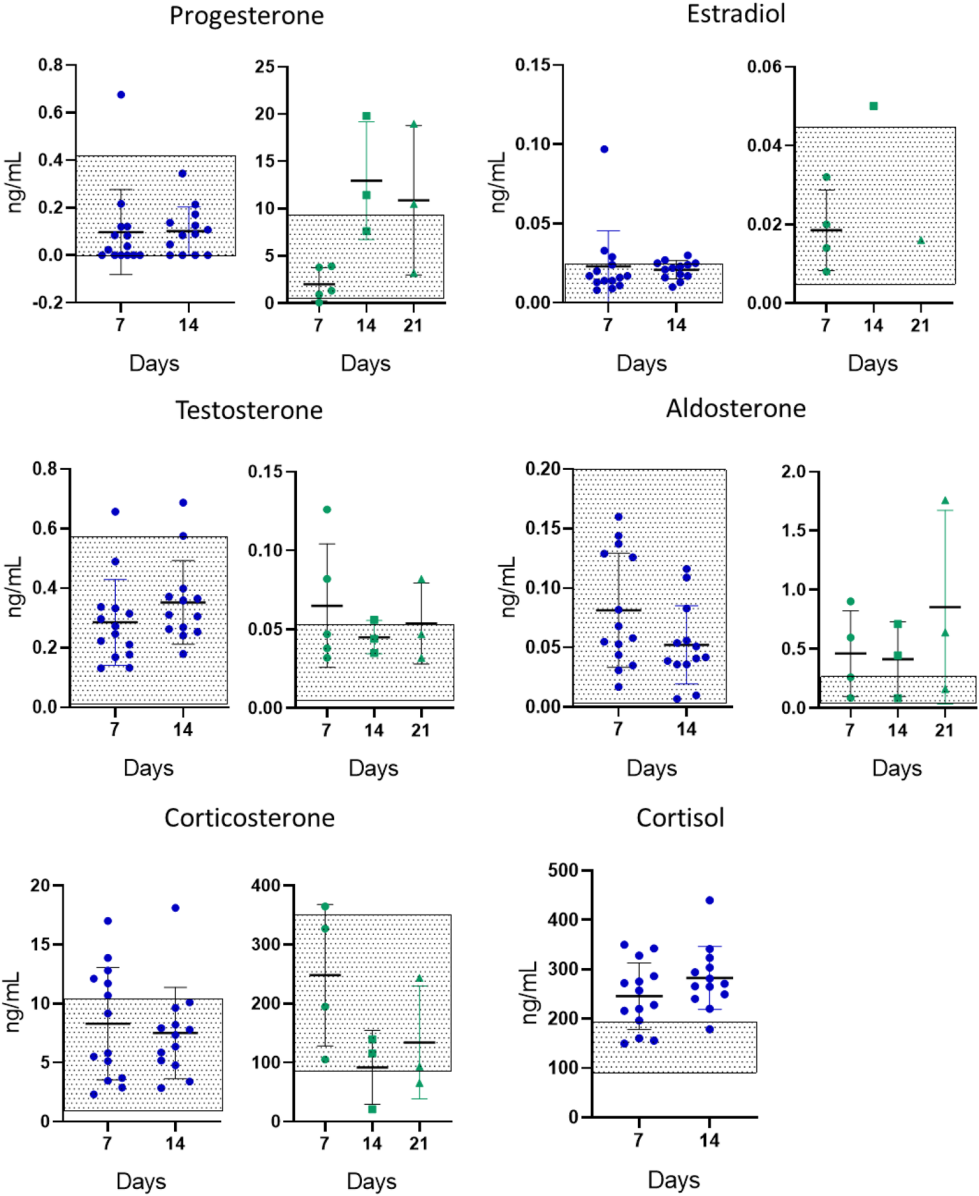
Comparison between the levels of the main steroid hormones in plasma from mifepristone-treated patients vs mifepristone-treated mice. Levels of steroid hormones (*X̄* ± s.d.) in mifepristone-treated patients after 7 or 14 days of treatment (left graphs in blue) *vs.* those observed in mice treated with mifepristone pellets for 7, 14, or 21 days (right graphs in green). The values that fall in the dotted areas correspond to values from the untreated patients or the untreated mice respectively. Progesterone levels were higher in mifepristone-treated mice for 14 days compared to those treated for 7 days. Data were analyzed using Kruskal–Wallis test. *P < 0.05. The statistical significance of changes observed in patients compared to control values is shown in Table 1. A full color version of this figure is available at https://doi.org/10.1530/ERC-23-0238.

We then evaluated the profile of 14 steroids in mifepristone-treated mice after 7, 14, or 21 days of pellet implantation (Table 2). In this case, the only statistically significant change was that of progesterone, in which a late increase after 14 or 21 days of treatment was observed. A similar trend was observed with the precursor pregnenolone and with aldosterone; the values of treated mice vs control mice only showed statistical significance if the values of all treated mice were grouped together (*P* < 0.05). The trend of an increase in the levels of aldosterone could be the product of the apparent decrease in corticosterone levels observed in mifepristone-treated mice. In Fig. 4, we show the levels of the main steroids comparing changes found in mifepristone-treated patients and in treated mice. In this figure, the steroid levels detected in untreated patients or in untreated mice are plotted as square boxes. It can be noticed that whereas in postmenopausal patients the most important potential side effects could be driven by the increase in corticoid-related steroids, this did not occur in mice in which low mifepristone levels were detected. Progesterone-related hormones increased in treated mice, probably counteracting mifepristone’s antiprogestin effects. Importantly, no increases in glucocorticoid steroids were evident in mice treated with mifepristone pellets at doses that may induce tumor regression.

**Table 2:** Steroid hormone profile in mice treated with daily doses or with mifepristone pellets.

Mifepristone administration	Control	12 mg/kg/day		Pellet 6mg			*P*^a^
	No drug (*n* = 9)	Day 2 (*n* = 3)	Day 7 (*n* = 3)	Day 7 (*n*= 5)	Day 14 (*n* = 3)	Day 21 (*n* = 3)	
Steroid	**Median (range), nmol/L**						
Mifepristone	–	69 (47–112)	146 (19.6–161.4)	20 (4.8–30)	32.9 (25.8–34.3)	23.3 (14.9–27.3)	–
Pregnenolone	0.9 (0.09–49.3)	2.3 (1.4–3.2)	5.2 (0.04–8.0)	2.8 (0.13–4.64)	4.2 (2.2–11.6)	5.3 (2.9–9.1)	ns
11-dehydrocorticosterone	8.9 (2.0–26.6)	0.9 (0.6–3.9)	2.5 (0.2–6.2)	9.4 (5.7–17)	5.1 (1.0–6.3)	4.9 (3.1–8.5)	ns
11-deoxycorticosterone	1.16 (0.3–5.2)	0.3 (0.2–0.3)	1.2 (0.7–6)	2 (0.5–3)	1.4 (0.3–2.2)	1.9 (0.5–5.3)	ns
17-hydroxyprogesterone	0.09 (0.03–0.3)	0.09 (0.03–0.3)	0.02 (0.01–0.04)	0.1 (0.07–0.13)	0.03 (0.02–0.04)	0.02 (0.02–0.03)	ns
Aldosterone	0.4 (0.1–0.8)	0.05 (0.05–0.05)	0.2 (0.12–0.24)	1.2 (0.2–2.5)	0.03 (0.03–0.04)	0.2 (0.01–0.2)	ns
Androstenedione	0.09 (0.06–1.2)	0.4 (0.1–0.7)	0.24 (0.06–0.5)	0.2 (0.09–0.7)	0.2 (0.2–0.3)	0.2 (0.1–0.2)	ns
Corticosterone	665 (261–1053)	160 (111–263)	293.4 (83–776)	650 (304–1054)	333 (60.4–402)	270 (189–703)	ns
Estradiol	0.1 (0.02–0.2)	0.2 (0.05–0.2)	0.1 (0.07–0.12)	0.06 (0.03–0.12)	0.18 (0.18–0.18)	0.06 (0.06–0.06)	ns
Estrone	0.01 (0–0.02)	0.01 (0–0.02)	0.01 (0–0.01)	0.01 (0–0.01)	0 (0–0)	0 (0–0)	ns
Progesterone	0.9 (0.3–29.9)	0.34 (0.3–3.1)	87 (24–176)	4.2 (0.2–12.4)	36.4 (24.3–63)	33.4 (10–60)	*P* < 0.05
Testosterone	0.06 (0.03–0.9)	0.35 (0.08–0.4)	0.19 (0.04–0.43)	0.1 (0.06–0.51)	0.15 (0.1–0.2)	0.2 (0.1–0.3)	ns

^a^Kruskal–Wallis test.

**Figure 5:**
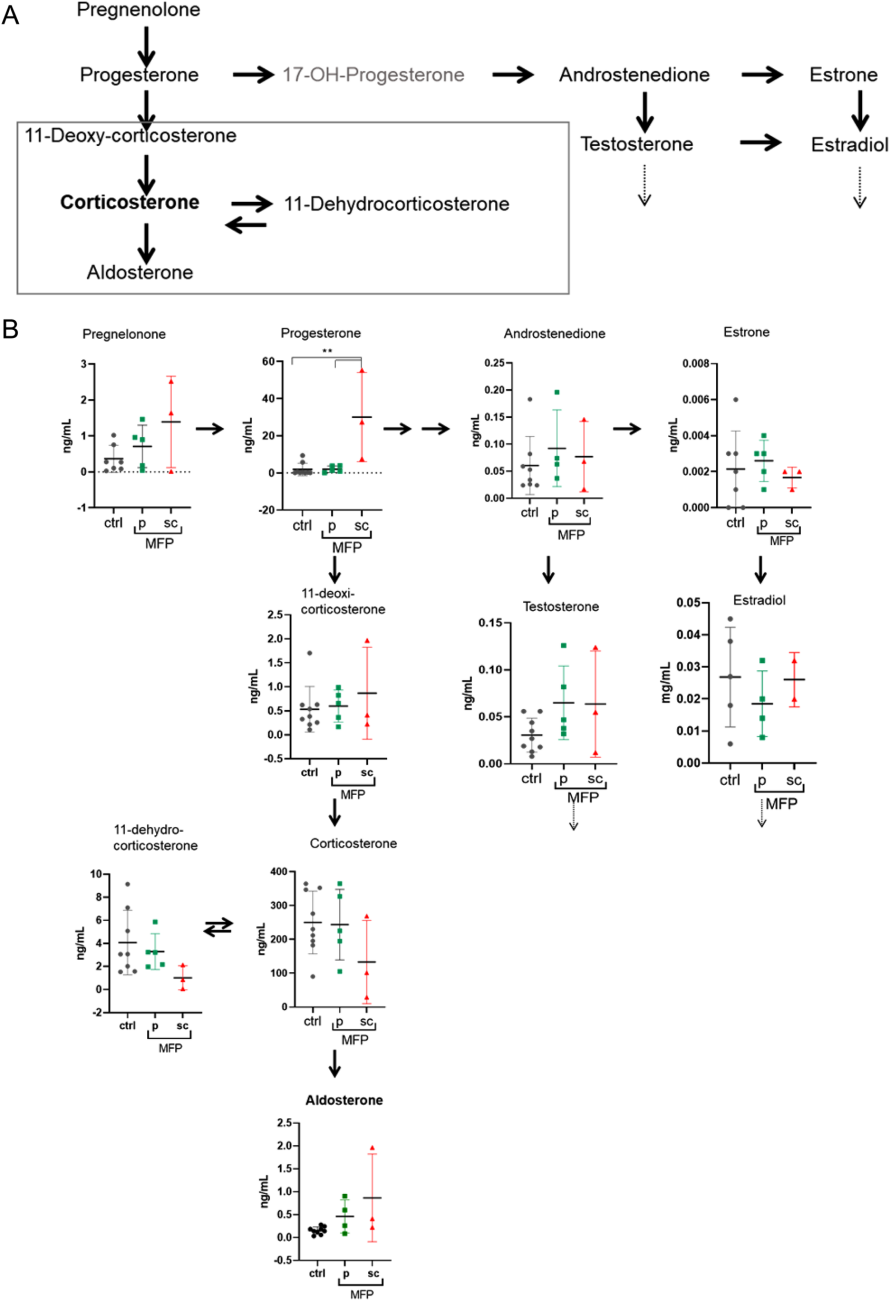
Changes in steroid hormone levels in mifepristone treated mice after 7 days of treatment with daily doses or pellets. (A) Mouse steroidogenic pathways: Scheme illustrating the synthetic pathways of corticoid and sexual related steroids. (B) Profile of different steroid hormones measured by Liquid extraction and LC-MS/MS in plasma from untreated female mice (ctrl) or those treated with mifepristone (MFP) pellets (6 mg) implanted subcutaneously or with daily doses (12 mg/kg body weight) subcutaneously (*X̄* ± s.d.). Data were analyzed using Kruskal–Wallis test. Progesterone levels were higher in mice treated with daily doses compared with controls or those treated with pellets; **P < 0.01. A full color version of this figure is available at https://doi.org/10.1530/ERC-23-0238.

Regarding the intrinsic comparison in the steroid profile between both administration schedules, the only significant difference was the increase in progesterone levels observed after 7 days of treatment with the daily doses (more than 15 times higher than control values). In pellet-treated mice, progesterone levels were similar at day 7 to those observed in control mice and as mentioned before, the increase was observed later, 14 or 21 days after pellet implantation (*P* < 0.05; Fig. 5). The data shown herein suggest that the mifepristone 6 mg handmade pellets may be a better choice than the daily injections.

## Discussion

In this study, we have shown that the therapeutic effect of mifepristone on mouse mammary carcinomas occurs at plasma concentrations much lower than those observed in the MIPRA trial, suggesting the possibility to diminish mifepristone doses in future clinical trials treating breast cancer patients. Lowering these doses could avoid the increase in plasma glucocorticoids levels maintaining the same therapeutic effects.

Mifepristone, in addition to being an antiprogestin, at higher concentrations behaves as an antiglucocorticoid (Gaillard *et al.* 1984) or as an antiandrogen (Song *et al.* 2004). In triple-negative breast cancer, mifepristone is also being tested in combination with chemotherapy to increase the efficacy (NCT02788981) (Skor *et al.* 2013). Besides, it has been proposed as a chemotherapeutic agent for advanced PR-negative cancer patients to improve the quality of life (Check *et al.* 2010). In this case, membrane progesterone receptors such as PGRMC1 may be involved, decreasing progesterone-induced blocking factor and thus increasing NK cells (Check & Check 2021). In both cases, the mechanisms proposed are unrelated to the canonical antiprogestin activity of the drug.

The doses used in the MIPRA trial have already been used in long-term clinical studies in which the authors had the intention to minimize the antiglucocorticoid effects (Grunberg *et al.* 2006, Ji *et al.* 2015). In fact for the treatment of Cushing disease, doses of 300–1200 mg/day are currently used (Karena *et al.* 2022). In the first clinical trial in which mifepristone was used to treat metastatic breast cancer patients, the 200 mg dose was selected considering that this dose would be equivalent to the daily 2.5 mg/kg dose administered in preclinical contraception assays in which an antiprogestin effect was assessed (Romieu *et al.* 1987). Furthermore, it was still selected in other trials involving breast cancer patients (Klijn *et al.* 2000). In our short-term study, increases in cortisol levels were observed in a similar range as those encountered by others (Romieu *et al.* 1987, Klijn *et al.* 1989). However, signs of biochemical side effects such as increases in glucose levels were not reflected in the laboratory analysis (Elia *et al.* 2022), suggesting that the cortisol increase after mifepristone treatment may be a compensatory effect that is still not clinically relevant in the 14-day treatment scheme. Interestingly, in patients treated with tamoxifen, an estrogen receptor modulator, the levels of cortisol and corticosterone also significantly increased, with values reaching in some patients near the micromolar range (Baumgart *et al.* 2014).

In humans, the time to maximum plasma concentration after oral administration of mifepristone in doses of 2–600 mg was 1–2 h after ingestion (Heikinheimo *et al.* 1987, Kekkonen *et al.* 1996). Remarkably, the maximum serum concentration was similar among doses between 100 to 800 mg and they remained in the micromolar range persisting up to 20 months after daily treatment (Heikinheimo *et al.* 1997). Only when doses lower than 25 mg were administered, the serum concentrations increased proportionally (Foldesi *et al.* 1996, Kekkonen *et al.* 1996). At higher doses, 94 to 99% of the drug binds to a high affinity binding protein identified as alpha-1-acid glycoprotein (AAG) and to albumin, suggesting that there is a threshold and that if surpassed, the amount of free mifepristone, that exerts the therapeutic effect, will still be the same. Thus, lower mifepristone doses may have a similar therapeutic impact than the higher ones.

The possibility that in mice lower mifepristone levels would be more bioactive because of different AAG and albumin serum concentrations (reviewed in (Heikinheimo 1997, Smith & Waters 2018, Islam *et al.* 2020)) seems unlikely, since the mass spectrometry method used herein measures bound and unbound mifepristone.

The mifepristone doses chosen to treat mouse mammary carcinomas were originally selected from studies carried out using the MXT mouse model, or DMBA-induced mammary carcinomas in rats (Bakker *et al.* 1990, Schneider *et al.* 1990). Then, in our laboratory we designed handmade Silastic pellets (Sahores *et al.* 2013). We have shown that the subcutaneous implantation of 6 mg pellets mimicked the daily dose inducing regression of tumors from the MPA-induced breast cancer model (Wargon *et al.* 2015), some of them being responsive even with pellets of 0.2 mg instead of the standard 6 mg dose (Sequeira *et al.* 2021).

In mice, the active glucocorticoid hormone is corticosterone, and no increases in corticosterone levels were observed in mifepristone-treated mice. In contrast to mifepristone-treated patients, an increase in progesterone-related steroids was observed. This might be a compensatory mechanism present in young female adult mice, not observed in postmenopausal women with nonfunctional ovaries. The increase in aldosterone may be due to an increase in the CYP11B2 enzyme activity that metabolizes corticosterone to aldosterone.

One weakness of this study is that we have compared serum levels of mifepristone between two different species; it is possible that the pharmacokinetics, pharmacodynamics, and possibly also the biological effects differ between both species. However, considering (i) that standard tamoxifen therapy is usually administered as daily tablets of 20 mg for 5–10 years to treat luminal breast cancer patients and the reported plasma tamoxifen levels range from 20 to 307 ng/mL (~545 nmol/L; MacCallum *et al.* (1997), Fotoohi *et al.* (2016), Bobin-Dubigeon *et al.* (2019)); (ii) that the antiprogestin telapristone acetate was administered at daily doses of 12 mg and therapeutic effects were observed in patients bearing plasma levels of 147 ± 111 ng/mL (Lee *et al.* 2019); (iii) that mifepristone has a higher affinity for PR than the natural ligands (Kd of mifepristone for the human or mouse PR is 1–2 × 10^−9^ mol/L (Heikinheimo *et al.* 1987, Montecchia *et al.* 1999)) and mouse tumors regressed completely with plasma levels lower than 20 ng/mL, it seems reasonable to propose the evaluation of lower mifepristone doses to treat luminal breast carcinomas with higher levels of PRA than PRB.

## Supplementary Materials

Supplementary Table 1. Lower and upper limit of detection of steroids measured by Mass Spect and expected range in premenopausal and postmenopausal women according to the data of Mayo Clinic Laboratories.

## Declaration of interest

Claudia Lanari and Paola Rojas have a patent (WO2013086379A2) regarding the use of antiprogestins in human breast cancer.

## Funding

The clinical study was funded by Agencia Nacional de Promoción de Ciencia y Tecnología, Ministerio de Ciencia y Técnica- Argentina, ANAPCYT (PIDC 2012-084) to C. Lanari, the analysis of the human plasma studies was funded by Fundación Sales to C. Lanari, and the analysis of the murine plasma samples was funded by ANAPCYT, PICT 2021-029.
