# Supplementary material for: Steroid profile in patients with breast cancer and in mice treated with mifepristone
Source: Endocr Relat Cancer. 2023 Dec 13;31(2):e230238. doi: 10.1530/ERC-23-0238 (PMC10762537; doi:10.1530/ERC-23-0238)
Supplement: Supplementary Table 1. Lower and upper limit of detection of steroids measured by Mass Spect and expected range in premenopausal and postmenopausal women according to the data of Mayo Clinic Laboratories. [file supplementary_table_1.pdf]

Supplementary Table 1. Lower and upper limit of detection of steroids measured by Mass Spect and expected range in premenopausal and postmenopausal women according to the data of Mayo Clinic Laboratories.

**Steroids**	**LLOD**[1]	**ULLOD**[2]	**Range premenopausal**[3] **ng/mL**	**Range postmenopausal**[3] **ng/mL**
11-deoxycorticosterone	0.013	2.5	<0.1	<0.1
11-deoxycortisol	0.013	2.5	<0.1	0.1-0.79
17-hydroxyprogesterone	0.025	5	<0.8 luteal, <2.8 follicular	<0.51
Aldosterone	0.013	2.5	<0.2 supine, <1.2 sitting	<0.2 supine, <1.2 sitting
Androstenedione	0.05	10	0.3-2	0.3-2
Corticosterone	0.05	250	0.5-15	0.5-15
Cortisol	2.5	500	30-230	a.m.: 70-250<br>p.m.: 20-140
Cortisone	0.125	25	5-13	5-13
5alpha-dihydrotestosterone	0.013	2.5	<0.3	≤0.128
Estradiol	0.013	2.5	0.015-0.35	<0.010
Estrone	0.013	2.5	0.017-0.2	0.007-0.04
Progesterone	0.05	250	<0.89 follicular, 1.8-24 luteal	≤0.20
Testosterone	0.05	10	0.08-0.6	0.08-0.6
21- deoxycortisol			-	<0.05 all ages
Pregnenolone			≥ 18 years: 0.33-2.48	≥ 18 years: 0.33-2.48
Estriol			-	<0.08
17-hydroxipregnenolone			-	0.31-4.5
Dehydroepiandrosterone			<8.0	<6.0

1: Lower limit of detection; 2: upper limit of detection 3: Data from Mayo Clinic Laboratories.
